# Biodistribution of Exosomes and Engineering Strategies for Targeted Delivery of Therapeutic Exosomes

**DOI:** 10.1007/s13770-021-00361-0

**Published:** 2021-07-14

**Authors:** Hojun Choi, Yoorim Choi, Hwa Young Yim, Amin Mirzaaghasi, Jae-Kwang Yoo, Chulhee Choi

**Affiliations:** 1 ILIAS Biologics Incorporated, 40-20, Techno 6-ro, Yuseong-gu, Daejeon, 34014 Republic of Korea; 2 Department of Bio and Brain Engineering, KAIST, 291 Daehak-ro, Yuseong-gu, Daejeon, 34141 Republic of Korea

**Keywords:** Exosome, Biodistribution, Pharmacokinetics, Targeted delivery

## Abstract

Exosomes are cell-secreted nano-sized vesicles which deliver diverse biological molecules for intercellular communication. Due to their therapeutic potential, exosomes have been engineered in numerous ways for efficient delivery of active pharmaceutical ingredients to various target organs, tissues, and cells. In vivo administered exosomes are normally delivered to the liver, spleen, kidney, lung, and gastrointestinal tract and show rapid clearance from the blood circulation after systemic injection. The biodistribution and pharmacokinetics (PK) of exosomes can be modulated by engineering various factors such as cellular origin and membrane protein composition of exosomes. Recent advances accentuate the potential of targeted delivery of engineered exosomes even to the most challenging organs including the central nervous system. Major breakthroughs have been made related to various imaging techniques for monitoring in vivo biodistribution and PK of exosomes, as well as exosomal surface engineering technologies for inducing targetability. For inducing targeted delivery, therapeutic exosomes can be engineered to express various targeting moieties via direct modification methods such as chemically modifying exosomal surfaces with covalent/non-covalent bonds, or via indirect modification methods by genetically engineering exosome-producing cells. In this review, we describe the current knowledge of biodistribution and PK of exosomes, factors determining the targetability and organotropism of exosomes, and imaging technologies to monitor in vivo administered exosomes. In addition, we highlight recent advances in strategies for inducing targeted delivery of exosomes to specific organs and cells.

## Introduction

To maintain organisms’ homeostasis, intercellular communication is the key event to control multiple biological processes including mediator secretion, cellular proliferation, differentiation, and apoptosis. Cells located remotely communicate each other via soluble factors including neurotransmitters, hormones, cytokines/chemokines, lipid mediators, and extracellular vesicles (EVs) [1–3]. EVs, cell-secreted natural nanoparticles, are classified into three subtypes including exosomes, microvesicles, and apoptotic bodies, which exhibit different biological characteristics in terms of biogenesis, content, morphology and size (exosomes: 30 ~ 200 nm, microvesicles: 100 ~ 1,000 nm, and apoptotic bodies: 1 ~ 5 μm) [3, 4]. Exosomes are single-membrane lipid bilayer vesicles generated either by vesicle budding into endosomes that mature into multivesicular bodies or by direct vesicle budding from the plasma membrane [5]. Exosomes are secreted by all living cell types and have been found in various body fluids such as plasma, urine, saliva, semen, and breast milk [6–11]. Microvesicles (or ectosomes) are formed by direct outward budding of the plasma membrane with size typically ranging from 100 to 1000 nm [12]. Apoptotic bodies are relatively larger lipid vesicles released by dying cells which contain fragments of apoptotic cells such as micronuclei, chromatin remnants, and intact organelles [13]. EVs have traditionally been defined and sorted based on their different densities and sizes which enables separation by various methods such as differential centrifugation, filtration, and size exclusion chromatography [14]. It should be noted, however, that due to the overlapping size and density between EVs such as exosomes and microvesicles, current EV isolation techniques have limitation regarding precise purification without completely excluding other groups of EVs. Within the EVs, microvesicles and exosomes are considered as delivery vehicles of diverse biological molecules for intercellular communication including delivery of nucleic acid (*e.g.* DNAs, RNAs), proteins, lipids, and carbohydrates. Of note, in vivo circulating exosomes isolated from body fluids (*e.g.* urine and blood) carry biological materials (*e.g.* proteins and nucleic acid) and represent current physiological conditions, which suggests the diagnostic value of exosomes as novel biomarkers for multiple pathophysiological conditions including cancer [15, 16].

Due to their biological and functional characteristics, the therapeutic potential of exosomes is also being investigated as either natural or engineered form for different therapeutic purposes, including drug delivery tools, biological targeting agents, and vaccination [17–22]. Naturally produced exosomes inherit physiological characteristics of originated cells. When treated in vivo, exosomes show comparable potency with better safety profile compared to the original cell therapy, which suggests their potential use as cell‐free therapeutics. Numerous efforts have been made to expand the use of exosomes in diverse therapeutic areas via either engineering exosome or exosome-producing cell for loading active pharmaceutical ingredients (API) cargos and for exosome targeting to specific tissues/cells. With various therapeutic exosome platform technologies, there are about 20 biotech companies around the world developing exosome therapeutics, some of which are already moving on to clinical stage of development [23–25].

Here, we will review the current knowledge of biodistribution and PK of systemically administered exosomes, and various active targeting strategies to improve target specificity with better clinical outcome.

## Biodistribution and pharmacokinetics (PK) of in vivo administered exosomes

Numerous biocompatible and nontoxic nanoparticles have been employed as drug delivery systems including liposomes, polymeric nanoparticles, and exosomes. The biodistribution and PK profile of nanoparticles represent the in vivo behavior of administered nanoparticles and determining these two parameters are the key for successful nanoparticles-mediated novel therapeutics development. Whereas this review paper is mainly focused on exosomes, readers can find recent advances regarding other EVs, such as microvesicles, on review papers cited here [12, 26].

Focusing on exosome therapeutics, the major tissues distribution of systemically administered exosomes generally include liver, spleen, kidney, lung and gastrointestinal tract, which can be altered by various factors such as cellular origin of exosomes, exosomal membrane composition (e.g., protein, lipid, and glycan) and pathophysiological condition of host [27–35]. Exosome engineering for targeted delivery of therapeutic exosomes to various tissues including brain, placenta, heart, spinal cord, and cartilages is also being investigated [36–41]. Once the exosomes are administered systemically, they show rapid clearance from the blood with less than a few minutes of half-life in the circulation of healthy animals, which is primarily due to the rapid clearance by circulating phagocytic cells including macrophages and neutrophils [29, 32, 42, 43]. In contrast with the blood PK, exosomes display prolonged retention in the tissues such as the liver and spleen, showing sustained retention longer than 24 h [27, 29, 32]. Nonetheless, careful interpretation is needed for analyzing the tissue PK of exosomes, since most exosome imaging techniques utilize methods to label the lipid bilayer of exosomes with various imaging dyes which may lead to tracking of the cell-ingested phospholipids and not the exosome itself.

## Factors modulating biodistribution and PK of in vivo administered exosomes

**Fig. 1:**
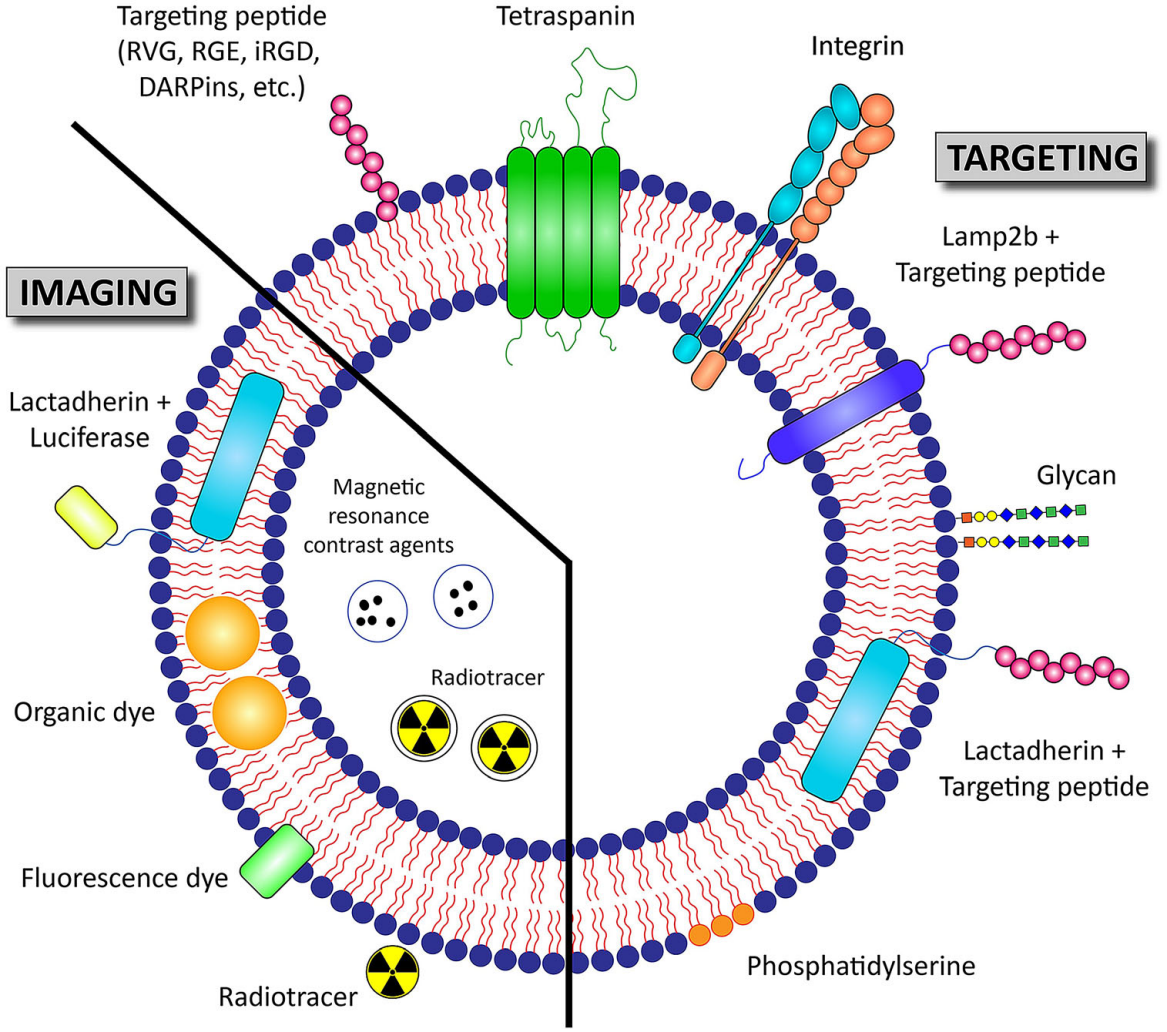
Targeting and biodistribution/PK analysis strategies of exosome therapeutics. Targeting of exosomes to specific organs or cells could be achieved via modification of the composition of exosomal membrane proteins including tetraspanins and integrins. Exosomal surface engineering by displaying targeting peptides conjugated with exosomal membrane-associated domains such as lysosome-associated membrane glycoprotein 2b (Lamp2b) or C1C2 domain of lactadherin (LA) is another approach for active tissue targeting. Both glycan and lipid compositions of exosomal membrane also contribute to the biodistribution of administered exosomes. Biodistribution/PK analysis of administered exosomes can be conducted via various exosome labeling methods (i.e., bioluminescence, fluorescence, and radio isotope-labeling methods)

Recent studies begin to identify molecules displayed on the exosomal membrane which determine their cellular or organ tropism (Fig. 1) [44, 45]. If the molecular mechanisms generating target cell tropism of exosomes is fully decoded, the potential of exosomes as a therapeutic vehicle would be greatly expanded, especially to the most challenging disease areas including the central nervous system (CNS) related diseases [46]. In this part, various factors that determine biodistribution and targetability of exosomes are discussed.

### Cellular origin

One of the main factors that determine the biodistribution of exosomes is its cellular origin. Exosomes from different cellular sources were observed to have an asymmetric biodistribution [27], and subsequent studies found an inclination that exosomes tend to have different tropism based on their cells of origin, which could be further utilized for organ-targeted delivery. For instance, targeted delivery of exosomes to the brain could be achieved by using neural stem cell (NSC)-derived EVs, as it showed preferential brain targeting compared to mesenchymal stem cell (MSC)-derived EVs in a murine stroke model [47]. Tumor-derived exosomes were shown to be efficient in targeting its parental tumor for delivering anti-cancer drugs [29, 30, 48–50]. In a HT1080 xenograft mouse model, systemically injected HT1080-derived exosomes were targeted more efficiently to HT1080 tumor burden compared to HeLa-derived exosomes, with HT1080-derived exosomes delivered twice as much as HeLa-derived exosomes [30]. In zebrafish model, exosomes derived from either brain endothelial cells or brain tumor cells crossed the blood–brain barrier (BBB) and successfully deliver anti-cancer drugs to the brain tumors, with 4 nl of 0.2 mg/ml doxorubicin loaded in 200 μg/ml of brain endothelial cell-derived exosomes inhibiting expression of VEGF RNA more than half compared to doxorubicin-only injected brain tumor model of zebrafish [48]. Moreover, prostate cancer cell lines LNcaP- and PC-3-derived EVs loaded with Paclitaxel (PTX) have been shown to be effective carriers for delivering PTX to their parental cells [49]. However, Smyth et al. suggested that tumor-derived exosomes showed the tumor targeting property only when the exosomes were injected locally into the tumors [29]. In their study, they observed a minimal tumor targeting of systemically injected breast and prostate tumor-derived exosomes [29]. Jung et al. also showed that hypoxic cancer cell-derived exosomes are targeted to hypoxic cancer cells only in vitro but not in vivo [50]. More in-depth mechanistic studies are required regarding targeting ability of tumor cell-derived exosomes to their parental cancers. Even with tumor-targeting benefits for utilizing tumor-derived exosomes, they may have safety issues when administered systemically: tumor-derived exosomes may deliver tumorigenic factors to healthy cells and moreover, promote tumor metastasis by initiating pre-metastatic niche formation in healthy tissues [51–53]. Therefore, utilizing tumor-derived exosomes for tumor therapeutics may not be feasible. Instead, mechanistic insight for understanding tumor tropism of tumor-derived exosome can be applied to design targeting approach for tumor therapeutics with exosomes.

### Membrane composition of exosomes (e.g., proteins, lipids, and glycans)

Cellular or organ targeting of exosomes is influenced by various membrane compositions of exosomes, such as proteins, lipids, and glycans. Membrane protein composition of exosomes is determined by their cellular origin as well as the physiological state of parental cells during exosome biogenesis [54]. For instance, exosomes originated from antigen-presenting cells, including dendritic cells, macrophages, and B cells, tend to display immune regulatory proteins and antigens similar to that of their parental cells [54]. Exosomes from mature dendritic cells were found to express mature phenotype markers such as major histocompatibility complex (MHC) class I, class II molecules, CD40, CD86 and ICAM-1/CD54 [55]. Similarly, exosomes released by B cells or T cells carry B-cell or T-cell receptor subunits, respectively [56, 57], and those derived from natural killer cells contain the NK cell marker CD56 [58], which partially resemble the features of the originated cells.

The major proteins that constitute the exosomal membranes are proteins such as tetraspanins (*e.g.,* CD9, CD63, CD81, CD82), integrins and MHC molecules, of which various composition of these proteins could influence organotropism of exosomes [5]. For instance, exosomes expressing integrin α_6_β_4_ and α_6_β_1_ are targeted to laminin-enriched lung microenvironments, especially to the S100-A4-positive fibroblasts and surfactant protein C-positive epithelial cells in the lungs [59]. In contrast, exosomes displaying integrin α_v_β_5_ preferentially interacts with fibronectin in the liver microenvironments, which is specifically targeted to F4/80 positive Kupffer cells [59]. Also, Qiao et al. identified eight different integrins (integrin αv, α3, α5, α6, β1, β4, β5, β6) in tumor-derived exosomes by proteome profiler array with receptor proteins, suggesting that these integrins contribute to the tumor tropism of tumor-derived exosomes [30]. Tetraspanins, which are abundant on the membrane of exosomes, also contribute to the organotropism of exosomes by forming a complex with other tetraspanins and integrins: exosomes with tetraspanin Tspan-8 and integrin α_4_ complex were readily targeted to endothelial and pancreas cells [60]. CD47 is the ligand for signal regulatory protein alpha (SIRPα), which upon binding initiates the ‘don’t eat me’ signal that inhibits phagocytosis [61]. Kamerkar et al. showed that CD47 expressed on the exosomes mediated protection from phagocytosis by monocytes and macrophages, which showed that engineering surface of exosomes with CD47 could induce prolonged circulation time [62].

The lipid and glycan composition of the surface of exosomes may also contribute to tissue tropism by modulating cellular uptake of exosomes [33, 63]. In vivo administered exosomes are rapidly up-taken by circulating phagocytic cells within several minutes after systemic administration [42, 43], and Matsumoto et al*.* found that the rapid uptake of intravenously administered B16-BL6 melanoma cell-derived exosomes by macrophages is mediated via recognizing negatively charged phosphatidylserine (PS) displayed on the membrane of exosomes [33]. Exosomal uptake could also be mediated by glycans on the membrane of exosomes. The uptake of glioblastoma (GBM) cell-derived EVs to the recipient GBM cells were shown to involve a triple interaction between the chemokine receptor CCR8 on the cells, glycans exposed on EVs and the soluble ligand CCL18, which in turn promoted GBM cell proliferation and resistance to the alkylating agent temozolomide [63].

### Pathophysiological conditions of host

The biodistribution and PK parameters of exosomes could be affected by the pathophysiological conditions of host. Grange et al. observed the biodistribution of MSC-derived EVs in a model of acute kidney injury (AKI) after intravenous injection and found significant accumulation of EVs in the kidney of AKI-induced mouse 15 min after exosome injection, whereas accumulation of EVs in kidney appeared 5 h after injection in healthy mouse [64]. BBB crossing can also be achieved at certain pathological conditions. Under the circumstances of brain inflammation, Yuan et al. showed that macrophage-derived exosomes expressing LFA-1 and C-type lectin receptors can penetrate the BBB by interacting with inflamed brain microvascular endothelial cells, with showing over three times more accumulation of exosomes in brain of inflamed mice compared to healthy mice [65]. An in vitro trans-well assay study demonstrated that unmodified exosomes can cross the BBB through endocytosis by brain microvascular endothelial cells which occurred only under stroke-like, inflamed conditions induced by TNF-α [66]. Mirzaaghasi et al. investigated the biodistribution and blood PK of HEK293T cell-derived exosomes in sepsis-induced mouse. They found that substantial number of exosomes were delivered to the lung compared to healthy mouse after intravenous injection, of which more than 30% of exosomes were delivered to the lung in sepsis-induced mouse after 1 h of injection whereas almost none were detected in the lung of healthy mouse. Also, prolonged retention of exosomes in the blood circulation were observed due to liver dysfunction [28]. In other disease models, clearance of fluorophore labeled exosomes (10 nmol, intravenous) from blood in normal mice were 0.0054–0.0154 mL/min [67], whereas Gaussia luciferase (gLuc)-lactadherin (LA) labeled exosomes (5 µg, intravenous) in macrophage-depleted mice [42] and ^125^I labeled exosomes (4 × 10^5^ cpm, intravenous) in Parkinson’s disease mouse model [65] were 0.651 ± 0.157 mL/h and 0.016 mL/min, respectively.

## Imaging methods for determining biodistribution and PK of exosomes

Currently, bioluminescence and fluorescence imaging are the most commonly use methods for monitoring in vivo behavior of administered exosomes. However, with the recent technological advances for deep tissue penetration imaging, other clinical imaging methods including magnetic resonance imaging (MRI), positron emission tomography (PET) and single photon emission computed tomography (SPECT) are also being utilized for biodistribution and PK studies of exosomes (Fig. 1) [27, 62, 68–71].

### Bioluminescence and fluorescence imaging methods for determining biodistribution and PK of exosomes

Most of the methods evaluating in vivo characteristics of exosomes are conducted by labeling exosomes with various lipophilic fluorescent dyes or luminescent probes. Wiklander et al. [27] and Peinado et al. [72] used B16F10 murine melanoma cell-derived exosomes labeled with DiR and PKH67 and observed the distribution of exosomes in liver, lung, gastrointestinal tract, and bone marrow by intravenous injection. Grange et al*.* evaluated exosomes labeled with a near infrared dye (DiD) and observed significant accumulation of MSC-derived exosomes in kidney of AKI-induced mouse compared to a normal mouse [64]. Saari et al. observed cancer targeting of prostate cancer cell-derived exosomes by labeled with both DiD and OG-Paclitaxel (PTX) via direct incubation method [49]. Labeling method for exosomal surface proteins such as tetraspanins also was used to evaluate exosome distribution at the cellular level. Suetsugu et al. used green fluorescent protein (GFP) tagged CD63 to determine breast cancer cell-derived exosomes targeting to the stroma at metastatic sites during the metastatic process [53].

One of the main advantages of luminescence compared to fluorescence labelling method is higher signal-to-noise ratio since the light is not emitted from exogenous light source. Tissue distribution of exosomes from various cellular origins have been studied using a fusion protein of gLuc and LA, taking advantage of the feature that the C1C2 domain of LA could fuse with the membrane of exosomes [73]. Also, Lai et al*.* used a fusion protein of gLuc and transmembrane domain of platelet-derived growth factor receptor (PDGFR) for in vivo tracing the exosomes [74].

### Radio-labeling and magnetic resonance imaging methods for determining biodistribution and PK of exosomes

For real-time quantitative monitoring of exosomes in deep organs where detection of fluorescence or luminescence labeling is not possible, radioisotope labeling showed higher contrast and resolution. González et al. tracked milk-derived exosomes labeled with ^99m^Tc radiotracer and evaluated the distribution by SPECT in different administration routes such as intravenous, intraperitoneal injection and intranasal instillation [75]. They found longer circulation of exosomes injected by intraperitoneal route than those injected by intravenous route. Moreover, Faruqu et al. labeled exosomes by both intraluminal and membrane conjugation of ^111^In without engineering originating cells [32]. Membrane labeling method showed higher efficiency and radiochemical stability compared to the intraluminal labeling method, and 56%, 38% and 3% of in vivo administrated exosomes accumulated in the liver, spleen, and kidney. Radioisotope labeling of exosomes could also make the highest contrast and resolution through PET imaging. Jung et al. successfully labeled mouse breast cancer-derived exosomes with ^64^Cu (or ^68^ Ga) and visualized by PET imaging [76]. Also, exosomes could be labeled with magnetic resonance contrast agents (MRI contrast agents) (e.g., superparamagnetic iron oxide nanoparticles (USPIO)) by methods such as electroporation and co-incubation [77–79]. A fusion protein of ferritin heavy chain (FTH1), an MRI contrast agent, conjugated with lactadherin could also be used to label exosomes for MRI imaging [80]. Although MRI has lower sensitivity compared with radioisotope-based imaging, MRI has comparative advantages that it harbors no risk of radiation burden and provides excellent soft tissue contrast with spatial resolution for deep tissues.

## Engineering strategies for active tissue targeting of therapeutic exosomes

To deliver therapeutic exosomes to the target cells or tissues, either passive or active targeting strategies of therapeutic exosomes can be utilized. Passive targeting of exosomes utilizes natural cellular tropism of exosomes, whereas active targeting achieves targeted delivery of exosomes through exosomal surface engineering by various technical approaches. There are two major strategies for active targeting of exosomes: one is non-genetic approach which directly engineers the surface of exosome with diverse exogenous modifications, and the other utilizes genetic approaches which non-directly engineers the exosomes via genetically modifying exosome-producing cells (Table 1) [81–83]. Here, we discuss more details for these two major technical approaches for exosomal surface engineering to achieve targeted delivery of therapeutic exosomes.

**Table 1:** Engineering strategies for inducing targeted delivery of therapeutic exosomes

Category	Classification		Method	Targeting moiety	Target cell/tissue	Reference
Direct engineering of exosomes	Chemical modification	Covalent modification	PEGylation	Aminoethyl anisamide-PEG (AA-PEG)	Sigma receptor overexpressing lung cancer	[89]
			Click chemistry	Neuropillin-1-targeting RGE peptide (RGERPPR)	Glioma	[90]
				c(RGDyK)	Cerebral vascular endothelial cells	[92]
		Non-covalent modification	Receptor–ligand binding	Superparamagnetic nanoparticle-transferrin conjugate	Cancer targeting under external magnetic field	[95]
			Electrostatic interaction	Cationic lipids/pH-sensitive fusogenic peptide	Enhance endocytosis-mediated cellular uptake	[96]
			Hydrophobic insertion	DOPE-NHS linker/cardiac homing peptide (CHP peptide, CSTSMLKAC)	Heart	[99]
	Physical modification	Exosome-liposome hybridization	Freeze-thawing	PEG-DOPS	HeLa cells	[100]
Indirect engineering of exosomes	Genetic modification of exosome-producing cells		GPI anchorage	Anti-EGFR nanobody	EGFR-expressing breast cancer	[107]
			Conjugation with C1C2 domain	Anti-Her2 scFv	HER2-expressing breast cancer	[109]
			Conjugation with Lamp2b	α_v_ integrin-targeting iRGD peptide	Breast cancer cell line	[110]
				NSCLC-homing peptide Tlyp-1	Lung cancer cell line	[111]
				RVG peptide	Brain/BBB	[35]
				HER2 targeting DARPins	HER2-expressing breast cancer	[112]
			Conjugation with CD63	Apo-A1	Hepatocellular carcinoma	[114]
			Cellular-nanoporation	U87-targeting CDX peptide, GL261-targeting CREKA peptide	U87 glioblastoma cell, GL261 glioma cell	[118]

### Direct engineering of exosomes

The surface of exosomes can be directly engineered via various chemical or physical modifications for inducing targetability of therapeutic exosomes. There are two major modification approaches for directly introducing targeting moieties to the exosomal surface: one is utilizing covalent attachments of targeting moieties such as “click chemistry”, and the other utilizes non-covalent methods [84]. Additionally, exosomes can be physically modified by inducing hybridization of exosomes with chemically modified liposomes for inducing specific cellular/tissue targeting.

#### Covalent modification of the surface of exosomes

Click chemistry utilizes covalent interactions between an alkyne and azide residue to form a stable triazole linkage, which can be applied to attach targeting moieties on the surface of exosomes in a variety of aqueous buffers including water, alcohols, and dimethyl sulfoxide (DMSO) [84–88]. PEGylation, which is a modification of exosome’s surface with branched polyethylene glycol (PEG), is one of the most common examples of chemical conjugation method that uses covalent attachments [89]. Exosomes modified with aminoethyl anisamide-PEG (AA-PEG) were shown to target the sigma receptor-overexpressing lung cancer by AA-PEG functioning as a targeting ligand for sigma receptor [90]. Jia et al. demonstrated that labeling exosomal membrane with neuropilin-1 (NRP-1) targeting peptide (RGE peptide) by click chemistry promoted glioma targeting and BBB penetration in orthotopic glioma models, since NRP-1 was reported to be overexpressed in glioma cells and tumor vascular endothelium [91, 92]. RGE-labeled exosomes showed nearly 1.5 times more accumulation in glioma of U251 tumor-bearing mouse 1 h after exosome injection and exhibited prolonged retention of exosomes in the tumor [91]. Similarly, c(RGDyK), a peptide which exhibits high affinity to integrin α_v_β_3_ which is expressed in reactive cerebral vascular endothelial cells after ischemia, was conjugated to the surface of MSC-derived exosomes by click chemistry for the treatment of stroke [93]. c(RGDyK)-labeled exosomes exhibited as high as 11-fold tropism to the lesion region of ischemic brain of mouse compared to scrambled c(RGDyK) peptide-labeled exosomes [93]. Also, it has been reported that Azide-Fluor 545 fluorescent molecules could be attached on the surface of exosomes via alkyne-based cross-linking reactions without altering the size and characteristics of exosomes [84]. However, the drawbacks of utilizing covalent bond is that covalent bonds are a very stable bond but mostly requires toxic chemicals for inducing the bonds, thus raising caution for applying covalent modification methods in therapeutics.

#### Non-covalent modification of the surface of exosomes

The membrane of exosomes can also be engineered via non-covalent methods such as receptor–ligand binding, electrostatic interaction, and hydrophobic insertion [94, 95]. Receptor–ligand binding approach was proposed by Qi et al., where transferrin was used to conjugate superparamagnetic magnetite colloidal nanocrystal clusters to the surface of reticulocyte-derived exosomes by binding to transferrin receptors expressed on the exosomes [96]. The approach utilizing electrostatic interaction to conjugate targeting moieties to exosomes involves interaction of cationic species with negatively charged functional groups on the exosomal membrane [95]. Nakase and Futaki employed this method to attach cationic lipids and a pH-sensitive fusogenic peptide (GALA) to the negatively charged membrane of HeLa-derived exosomes [97]. In turn, GALA expressing exosomes showed increased binding to the endosomal membrane after endocytosis to HeLa cells, which facilitated the intracellular delivery of cargos into the cytosol. However, the drawback of using cationic molecules is that it could induce defects in supported lipid bilayers of target cells by generating disruptions such as formation of holes, membrane thinning, and/or membrane erosion [95, 98]. Hydrophobic interactions could be applied for direct insertion of targeting moieties to the exosomal membranes. Attachment of siRNAs with lipid conjugates such as fatty acids, sterols, and vitamins by covalent conjugation induced efficient loading of siRNAs into EVs driven by the hydrophobicity of the lipid conjugates [99]. DOPE-NHS (1,2-Dioleoyl-sn-Glycero-3-Phosphoethanolamine-N-hydroxysuccinimide) is a hydrophobic chemical which can be used to conjugate targeting peptides into the membrane of exosomes. For targeting the heart, cardiac stem cell-derived exosomes were conjugated with cardiac homing peptide (CHP; CSTSMLKAC) via DOPE-NHS linker, resulting in retention of the exosomes within the heart [100].

#### Exosome-liposome hybridization

Exosome-liposome hybridization method could be used to optimize the properties of the exosome surface in order to reduce immunogenicity, promote colloidal stability, increase blood PK, and therefore enhance target cell uptake of in vivo administered exosomes [101]. Sato et al. developed a method to generate exosome-liposome hybrid by freezing exosomes and liposomes together in liquid nitrogen and then thawing at room temperature for 15 min [101]. The exosome-liposome hybrid enhanced membrane fusion with HeLa cells compared to original exosomes isolated from either RAW 264.7 macrophages or HeLa cells [90, 101]. The lipid charge of exosome-liposome hybrid also influences target cell uptake, as exosomes hybridized with neutral or anionic liposomes had a higher probability to be uptaken by carcinoma cell line than those hybridized with cationic liposomes [85, 87]. The drawback of this method is the risks of altering the integrity and direction of membrane proteins on the exosomes, thus weakening their biological functionalities [102].

### Indirect engineering of exosomes by modifying exosome-producing cells

**Fig. 2:**
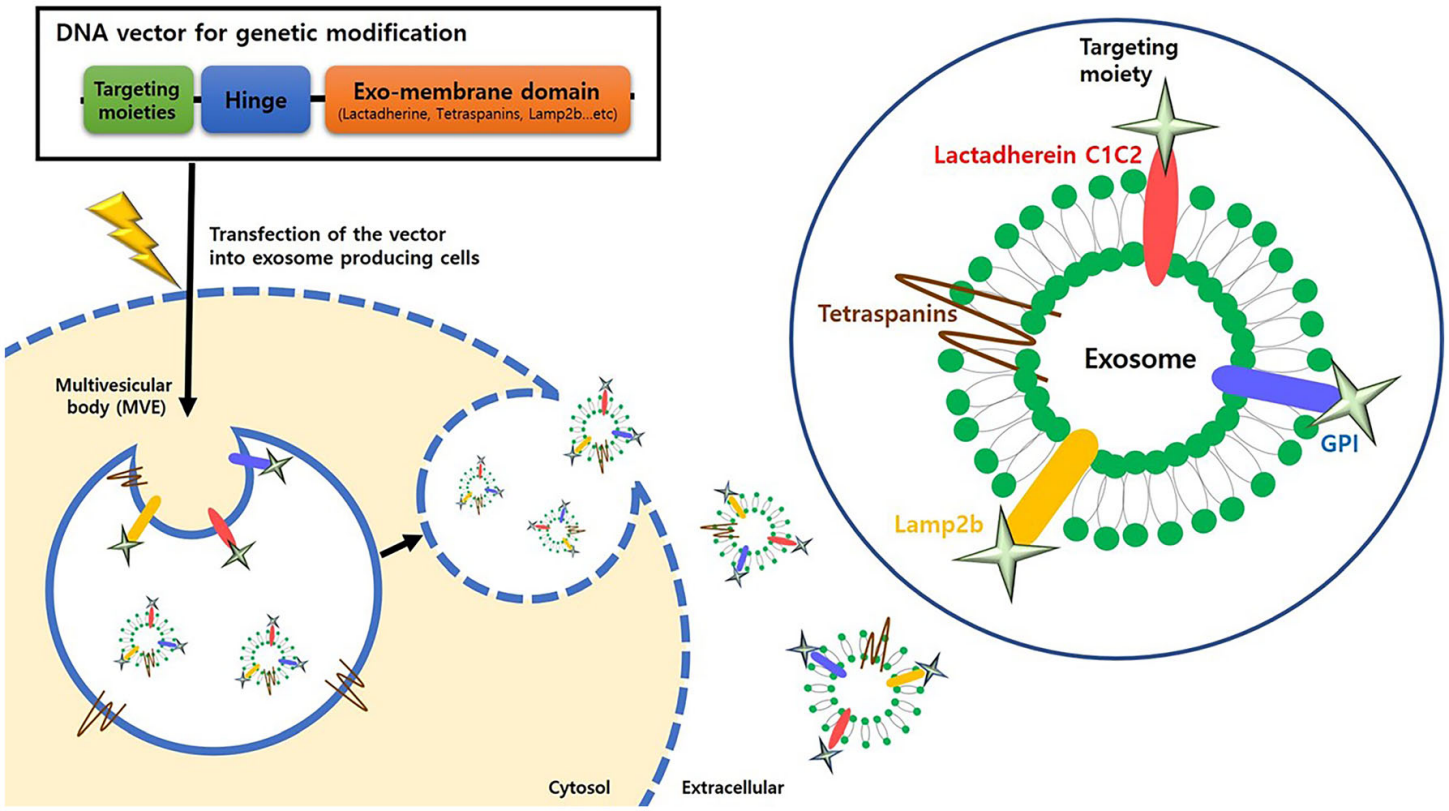
Schematic of genetic modification strategies for active targeting of exosomes. The vectors are designed to express the targeting moieties conjugated with exosomal membrane localizing domains, with the representative applications shown as follows: Tetraspanins, glycosylphosphatidylinositol (GPI), lysosomal-associated membrane protein 2 (Lamp2b), and C1C2 domain of LA. Cell/tissue-targeting peptides, receptors, and antibodies (scFv and nanobodies) are candidates of targeting moieties for specific tissue delivery. After the vectors are transfected into exosome-producing cells, the cells release the exosomes expressing targeting moieties on their surface

The surface of exosomes can be engineered indirectly via genetically modifying the exosome-producing cells, which holds several advantages over directly modifying exosomes in terms of expression yield and stability of targeting moiety displayed on engineered exosomes [103, 104]. The genetic modification of exosome-producing cells is achieved by transfecting genes expressing targeting moiety (*e.g.*, peptides, receptors and antibodies) which is fused with exosomal membrane components such as tetraspanins, Lamp2b, and C1C2 domain of lactadherin (Fig. 2) [103, 105]. The donor cells transfected with these vectors generate surface-modified exosomes expressing the targeting moieties via natural exosome biogenesis process. These exosomes produced from genetically engineered cells stably display introduced targeting moiety on their surface [106]. The applications of this exosome targeting strategy have been investigated in various diseases including cancer and CNS diseases [107]. Here, we discuss more details about this genetic engineering approach for targeting cancer and CNS.

#### Targeting cancer cells with indirect exosome modification approach

Recently, there has been considerable attentions in active targeting of therapeutic exosomes to various cancer cells (*e.g.*, breast cancer, NSCLC, HCC). To deliver therapeutic exosomes to cancer cells, targeting moieties which could bind to specific integrins or receptors expressed on cancer cells, such as antibodies and peptides, could be expressed on the surface of therapeutic exosomes with genetic modification approach. For instance, fusion proteins of anti-EGFR nanobodies conjugated with glycosylphosphatidylinositol (GPI)-anchor signal peptide were utilized to induce targetability of exosomes to EGFR-expressing tumor cells [108]. Exosomes are enriched in lipid raft-associated lipids and proteins, including GPI and GPI-anchored proteins, which enables exosomal surface labeling by anchorage to GPIs [109]. Exosome-producing cells were genetically engineered to express nanobodies fused at its C-terminus with GPI signal peptide derived from GPI-anchored protein decay-accelerating factor (DAF, also known as CD55) [108]. When expressed in cells, the DAF peptide conjugated with nanobodies were cleaved off by GPI transamidase enzymes, thereby driving direct conjugation of nanobodies to the GPI anchors [108]. In addition, C1C2 domain of lactadherin, which associates with the outer exosome membrane by interaction with phosphatidylserine, was used to express anti-Her2 single-chain variable fragments (scFv) on the surface of therapeutic exosomes [110]. Exosome-producing cells were genetically engineered to generate Her2-targeting exosomes by expressing the fusion protein of anti-Her2 scFvs fused at its C-terminus with the C1C2 domain of lactadherin, and N-terminus with a signal peptide directing the fusion protein into the secretory pathway to induce binding of fusion protein to the outer membrane of exosome, respectively [110]. Compared to non-engineered exosomes, these exosomes exhibited better cellular uptake by EGFR- or HER2-expressing breast cancer cells in vitro, showing approximately twofold and two ~ threefold higher accumulation, respectively [108, 110]. Also, α_v_ integrin-specific iRGD peptide fused with Lamp2b was over-expressed on the surface of exosomes produced from immature dendritic cells (iDCs), and these exosomes were loaded with doxorubicin as API via electroporation. Compared to non-engineered exosomes, these exosomes exhibited threefold better cellular uptake by human breast cancer cells in vitro and showed better anti-tumor efficacy in vivo with threefold decrease of tumor volume compared to tumor volume of non-engineered exosome-treated mouse [111]. Similarly, non-small cell lung cancer (NSCLC)-homing peptide, Tlyp-1, was conjugated with Lamp2b and displayed on the exosomal surface for targeted delivery of therapeutic exosomes to human lung cancer cells, showing twofold uptake of engineered exosomes to A549 NSCLC tumor cells. [103, 112]. Gomari et al. fused a HER2 targeting DARPins (designed ankyrin repeat proteins) to Lamp2b for targeting HER2 positive breast cancers which resulted in fourfold uptake of engineered exosomes to HER2 positive BT-474 breast cancer cells in vitro [113]. Apo-A1 was also conjugated with CD63, one of the tetraspanin markers of exosomes, for targeted delivery to HepG2 by utilizing Apo-A1 as a ligand for the scavenger receptor class B type 1 expressed on HepG2, which exhibited twofold increased uptake of engineered exosomes [114]. Gong et al. also developed a strategy to target triple-negative breast cancer (TNBC) by expressing a disintegrin and metalloproteinase 15 (A15) on the membrane of exosomes [115]. A15 binds to the integrin α_v_β_3_ in an RGD (Arg-Gly-Asp) dependent manner, which could target α_v_β_3_ overexpressing tumors such as melanoma, glioma and breast cancer [116–118]. Overall, expressing specific cancer targeting moieties on the surface of exosomes by conjugating with exosomal membrane-associated domains such as GPI, C1C2 domain, Lamp2b, and tetraspanins could serve as a promising strategy for active targeting of cancer cells for therapeutic exosomes.

#### CNS targeting with indirect exosome modification approach

Almost 98% of all drugs do not penetrate the BBB, and methods to deliver drugs through the BBB are actively being researched in biopharmaceuticals [46]. To achieve active brain targeting of exosomes, a 29-mer RVG peptide, a fragment of the polypeptide of Rabies Virus Glycoprotein (RVG), was incorporated into the exosomal surface by fusion with exosomal membrane protein Lamp2b [36]. Intravenous injection of RVG-tagged exosomes loaded with *GAPDH* siRNA specifically delivered the siRNA to neurons, microglia, oligodendrocytes in the brain, resulting in approximately twofold knockdown of *GAPDH* mRNA compare to non-treated mouse in vivo [36]. To produce large quantities of exosomes with brain targeting peptides and therapeutic cargoes, cellular-nanoporation method has been developed [119]. The system enables culture of monolayer of exosome-producing cells above the surface of chip containing an array of nanochannels (approximately 500 nm in diameter), which enable the passage of transient electrical pulses to shuttle plasmid DNA from the buffer into the attached exosome-producing cells with high yield. Two different peptides, a CDX peptide (FKESWREARGTRIERG) for U87 glioblastoma targeting or the peptide CREKA for GL261 glioma targeting, were inserted with a Flag epitope into the N terminus of CD47 to express on the surface of exosomes [119]. Utilizing exosomes containing *PTEN* mRNA loaded by cellular nanoporation, CDX-labeled exosomes showed approximately twofold higher accumulation in orthotopically implanted U87 glioma in nude mice and prolonged survival with a median survival of 49 days, compared with 37 days for non-targeted exosomes [119]. Also, *PTEN* mRNA-loaded, CREKA-labeled exosomes exhibited 1.5-fold higher accumulation in orthotopically implanted U87 glioma in C57BL/6 mice and prolonged survival with a median survival of 45 days, compared with 31 days for non-targeted exosomes [119].

## Summary and perspective

As biological messengers, exosomes deliver biological molecules between tissues/cells in both normal and pathophysiological conditions. Functionally, by delivering their contents including proteins, metabolites, and nucleic acids from donor cells into recipient cells, exosomes alter recipient cells’ biological responses. Exosomes can also transmigrate tissue barriers (e.g. BBB and placenta) with high biocompatibility, high yield capacity for intercellular cargo delivery and low immunogenicity. Due to these biological and functional characteristics, exosomes can be utilized as novel therapeutic platform for delivering various API cargos with specific tissue/cell targetability via multiple exosome engineering technologies.

Here, we reviewed the current knowledge for biodistribution and PK of systemically administered exosomes, factors influencing the targetability of exosomes, and technologies to determine administered exosomes’ in vivo fate. We also summarized technologies and strategies for inducing active targeting of exosomes to specific tissues/cells. These factors are essential for developing next-generation exosome therapeutics with clinical efficacy and safety. Although there are still significant technological huddles to overcome for developing clinical-grade exosomes, the efforts made by the leading companies developing exosome therapeutics will pave the way for conquering the scientific and technological challenges. In conclusion, we can position exosome as an attractive next-generation therapeutic platform to treat the various human diseases with medical unmet needs.
